# Integrated Analysis of Dysregulated lncRNA Expression in Fetal Cardiac Tissues with Ventricular Septal Defect

**DOI:** 10.1371/journal.pone.0077492

**Published:** 2013-10-16

**Authors:** Guixian Song, Yahui Shen, Jingai Zhu, Hailang Liu, Ming Liu, Ya-Qing Shen, Shasha Zhu, Xiangqing Kong, Zhangbin Yu, Lingmei Qian

**Affiliations:** 1 Department of Cardiology, The First Affiliated Hospital of Nanjing Medical University, Nanjing, People’s Republic of China; 2 Department of MICU, Nanjing Maternity and Child Health Care Hospital Affiliated to Nanjing Medical University, Nanjing, China; 3 Department of Pediatrics, Nanjing Maternity and Child Health Care Hospital Affiliated to Nanjing Medical University, Nanjing, China; 4 Department of Laboratory Medicine, Taizhou Maternity and Child Health Care Hospital, Taizhou, China; Harbin Institute of Technology, China

## Abstract

Ventricular septal defects (VSD) are the most common form of congenital heart disease, which is the leading non-infectious cause of death in children; nevertheless, the exact cause of VSD is not yet fully understood. Long non-coding RNAs (lncRNAs) have been shown to play key roles in various biological processes, such as imprinting control, circuitry controlling pluripotency and differentiation, immune responses and chromosome dynamics. Notably, a growing number of lncRNAs have been implicated in disease etiology, although an association with VSD has not been reported. In the present study, we conducted an integrated analysis of dysregulated lncRNAs, focusing specifically on the identification and characterization of lncRNAs potentially involving in initiation of VSD. Comparison of the transcriptome profiles of cardiac tissues from VSD-affected and normal hearts was performed using a second-generation lncRNA microarray, which covers the vast majority of expressed RefSeq transcripts (29,241 lncRNAs and 30,215 coding transcripts). In total, 880 lncRNAs were upregulated and 628 were downregulated in VSD. Furthermore, our established filtering pipeline indicated an association of two lncRNAs, ENST00000513542 and RP11-473L15.2, with VSD. This dysregulation of the lncRNA profile provides a novel insight into the etiology of VSD and furthermore, illustrates the intricate relationship between coding and ncRNA transcripts in cardiac development. These data may offer a background/reference resource for future functional studies of lncRNAs related to VSD.

## Introduction

The heart is extraordinary, not only in its complex ontogeny, architecture and uninterrupted contractility, but also in its ability to respond acutely to changing physiological and neuropsychological circumstances. Heart development requires precise temporal and spatial regulation of gene expression, in which the highly conserved modulation networks of transcription factors accurately control the signaling pathways required for normal cardiovascular development. Even slight perturbation of such programming during cardiogenesis can be catastrophic for the embryo or give rise to a spectrum of congenital heart defects(CHD)[1], which place enormous burdens on patients and families alike, and often stretch the limits of ethics, critical care medicine, and health budgets.

Therefore, the regulatory networks that control the development and adaptations of the heart have been under intense investigation[2]. Emphasis is now being placed on elucidating the complex interplay between the many hierarchical levels of gene regulation that give a network its dynamic properties and ultimately arranges cells into a myriad of precisely sculpted three-dimensional tissues and interacting organ systems. Due to the development of new molecular and developmental biologic techniques in the past decade, we have witnessed spectacular progress in elucidation of the molecular mechanisms of heart formation. In particular, several transcription factors (TFs) such as NKX2.5[3], Tbx5[4] and GATA4[5], have been identified as being essential for heart development. Nevertheless, the upstream regulators as well as interacting partners and downstream targets/effectors of the handful of TFs known so far to be linked to human CHD, remain largely unknown.

Recently, much interest has arisen in a heterogeneous class of small regulatory ncRNAs that directly affect the expression or function of protein-coding genes. These include microRNAs (miRNAs), endogenous small interfering RNAs, and PIWI-interacting RNAs[6]. MiRNAs, which represent the most extensively studied class of ncRNAs, have been proven to play key roles in heart development and are known as “fine-tuners” of cardiovascular system homeostasis. This is achieved by negative regulation of the expression of their target genes through post-transcriptional processes[7]. However, the study of lncRNAs, which comprise the bulk of the human non-coding transcriptome in the heart, is still in its infancy. LncRNAs are transcripts of at least 200 nucleotides transcribed from all over the genome, including intergenic regions, antisense, overlapping or intronic to protein-coding genes[8]. Many lncRNAs serve vital molecular functions, including structural or trafficking roles, controlling cell cycle, differentiation, and apoptosis, and serving as precursors for smaller RNAs. LncRNAs have a broad range of possible mechanisms of action, such as enhancer-like activity[8], establishment of repressive chromatin in genomic regions or entire chromosomes[9], intronic antisense transcripts capable of binding to histone modifiers thereby regulating the transcriptional output of the host gene[10], alternative splicing and other post-transcriptional RNA modifications that determine the activity of the genome[11].

A more sophisticated understanding of network structure and logic incorporating lncRNAs would impact profoundly on cardiovascular science and therapies and may provide unprecedented opportunities for intervention in disease progression.

Ventricular septal defect (VSD) is the most common form of CHD and among the most frequently observed congenital abnormalities [12]. However, so far lncRNAs related to VSD have not been reported. Recently, next generation transcriptome sequencing and microarray techniques have provided a method to delineate the entire set of transcriptional aberrations in a disease, including novel transcripts and non-coding RNAs not measured by conventional analyses[13]. Herein, using the comprehensive lncRNA microarray technique termed ‘‘Arraystar Human LncRNA Microarray v2.0’’, we investigated the dysregulated lncRNA profile by comparing the transcriptome of normal and VSD-affected cardiac tissue.

## Materials and Methods

### Preparation of fetal tissues

Fetal cardiac and other organ tissue samples were obtained from pregnant women who underwent induced abortion in Nanjing Maternity and Child Health Care Hospital. Experimental fetus enrollment criteria included: gestational age between 17 and 20 weeks; with ventricular sepal defect diagnosed by fetal echocardiography; especially excluding those fetuses with other common developmental defects. Normal Control (NC) fetuses of equivalent gestational age in weeks were obtained from pregnant women who underwent voluntary abortion due to private reasons. Finally, 10 VSD fetuses and 10 NC fetuses were selected. Sample collection was approved by The Nanjing Medical University Ethics Committee on research involving human subjects, and written informed consent was obtained from the parents in each case. All experiments were performed in compliance with the Helsinki Declaration and national laws.

### RNA extraction

To extract RNA, frozen tissues were ground into powder with mortar and pestle and resuspended in TRIzol reagent (Invitrogen, Carlsbad, CA, USA). RNA purification was performed on the RNA-containing aqueous phase with the RNeasy minikit (Qiagen, Valencia, CA, USA). RNA was then eluted with RNase-free water and treated with turbo DNase (Ambion) to remove contaminating DNA. Quantification and quality evaluation were performed with Nanodrop and Agilent 2100 Bioanalyzer (Agilent Technologies), respectively.

### Microarray analysis

Arraystar Human LncRNA Microarray v 2.0 is designed for the global profiling of human lncRNAs and protein-coding transcripts. Each transcript is represented by a specific exon or splice junction probe which can identify individual transcripts accurately. Positive probes for housekeeping genes and negative probes are also printed onto the array for hybridization quality control. Briefly, mRNA was purified from total RNA after removal of rRNA (mRNA-ONLY™ Eukaryotic mRNA Isolation Kit, Epicentre). Then, each sample was amplified and transcribed into fluorescent cRNA along the entire length of the transcripts without 3’bias utilizing a random priming method. The labeled cRNAs were hybridized onto the Human LncRNA Array v 2.0 (8 × 60 K, Arraystar). After having washed the slides, the arrays were scanned by the Agilent Scanner G2505C. Agilent Feature Extraction software (version 11.0.1.1) was used to analyze acquired array images. Quantile normalization and subsequent data processing were performed using the GeneSpring GX v11.5.1 software package (Agilent Technologies). Finally, four samples were hybridized; two biological replicates for each condition (cardiac tissues of VSD fetal and NC control, respectively). Differentially expressed lncRNAs with statistical significance were identified through Volcano Plot filtering. The threshold used to screen up or downregulated lncRNAs was fold-change >2.0 (*P* < 0.05).

### Quantitative real-time reverse transcription PCR

cDNA was synthesized from 1 μg total RNA using the AMV Reverse Transcriptase Kit (Promega, Madison, WI, USA). Real-time PCR was performed using the SYBR green method in an Applied Biosystems 7300 Sequence Detection System (ABI 7300 SDS; Foster City, CA, USA), following the manufacturer’s protocols. The PCR conditions included a denaturation step (95°C for 10 minutes), followed by 40 cycles of amplification and quantification (95°C for 15 seconds, 60°C for 1 minute). Relative gene expression levels were quantified based on the cycle threshold (Ct) values and normalized to the reference gene glyceraldehyde 3-phosphate dehydrogenase (GAPDH). Each sample was measured in triplicate, and the gene expression levels were calculated by the 2 – ^ΔΔCt^ method. The sequences of the primers used are shown in Table S1.

### GO and pathway analysis

Previous studies have shown that mammalian lncRNAs are preferentially located next to genes with developmental functions. For each lncRNA locus the nearest protein-coding neighbor within <100 kb was identified. For antisense overlapping and intronic overlapping lncRNAs, the overlapping gene was identified. Pathway and GO analyses were applied to determine the roles of these closest coding genes in biological pathways or GO terms. GO analysis was applied to analyze the main function of the closest coding genes according to the GO database which provides the key functional classifications for the National Center for Biotechnology Information (NCBI)[14]. Generally, Fisher’s exact test and v2 test were used to classify the GO category, and the false discovery rate (FDR) was calculated to correct the *P*-value. Gene networks and canonical pathways representing key genes were identified using the curated IPA (Ingenuity Pathway Analysis) database according to KEGG, Biocarta, and Reatome, as previously described [15]. Fisher’s exact test and v2 test were used to select the significant pathway, and the threshold of significance was defined by the *P*-value and FDR.

### Sequence conservation analysis

One of the best strategies known for finding functional sequences is to look for sequences that are conserved across species. An improved approach is to compare multiple species with different evolutionary distances to compute general conservation metrics for a genomic region. We separately downloaded the base-by-base phastCons scores and the phastCons-predicted conserved elements across the 46 vertebrates, including the 33 placental mammal and the 10 primate subsets of species in these 46 vertebrates from UCSC (http://genome.ucsc.edu/). The phastCons scores can be interpreted as probabilities that each base is in a conserved element, given the assumptions of the model and the maximum-likelihood parameter estimates [16]. We calculated the base conservation in each lncRNAs using the criteria that phastCons scores of not less than 0.5 indicate high conservation. The global conservation was assessed by calculating the median phastCons score of each category of dysregulated lncRNAs.

### Bioinformatics analysis

Non-coding regions harbor transcriptional regulatory elements; however, it can be challenging to distinguish these using only the primary sequences as a guide. The protocol used in this study involved mapping of various epigenetic phenomena to aid in the identification of non-coding regulatory elements. Tri-methylation of lysine 4 (H3K4me3) tends to mark promoters, whereas mono-methylation of the same lysine subunit (H3K4me1) tends to mark enhancers[17]. Acetylation of lysine 27 (H3K27ac) also associates with enhancers; however, it appears to have some specificity for those that are active rather than those that are merely “poised” for activity[18]. Transcription factor occupancy of a particular sequence can be another useful indicator of regulatory function, especially in combination with the aforementioned marks. Typically, transcription factor binding sites are less broad and can thus be more helpful in precisely mapping the boundaries of a regulatory element. In addition, regulatory sequences are often hypersensitive to DNase treatment when applied to native chromatin. The ENCODE Integrated Regulatory track on the UCSC genome browser makes it easy to scan for all such regions described above. The methods used to gather data for this track are described in an extensive publication by the ENCODE Project Consortium (2011). Furthermore, we made predictions of the TF motifs which combined with lncRNA loci using online sources (*http://motifmap.ics.uci.edu/*).

In order to explore the potential targets of lncRNA, we analyzed the RNA-protein interaction of lncRNA and corresponding TFs based on catRAPID algorithm, a free resource which can be obtained online (*http://service.tartaglialab.com/page/catrapid_group*). The experimental determination of ribonucleoprotein (RNP) complexes is a slow and difficult process and the number of experimentally determined structures of RNP complexes is still rather scarce. In view of this, computational predictions of RNP complex structures would greatly facilitate the investigation of protein-RNA interactions and their molecular function. Through the calculation of secondary structure, hydrogen bonding and van der Waals contributions, catRAPID predicts protein-RNA interaction propensities with great accuracy (up to 89% on the ncRNA-protein interaction database, NPinter).

### Statistical analysis

Expression levels of lncRNAs were compared by the paired sample *t*-test. The χ^2^, Fisher exact probability, and Student's *t*-test were used for comparisons between groups. Data are expressed as the mean ± standard deviation from at least three independent experiments. All *P*-values were two-sided and obtained by using the SPSS 16.0 software package (SPSS, Chicago, IL, USA). A value of *P* < 0.05 was considered statistically significant.

## Results

### Profile of microarray data

**Figure 1:**
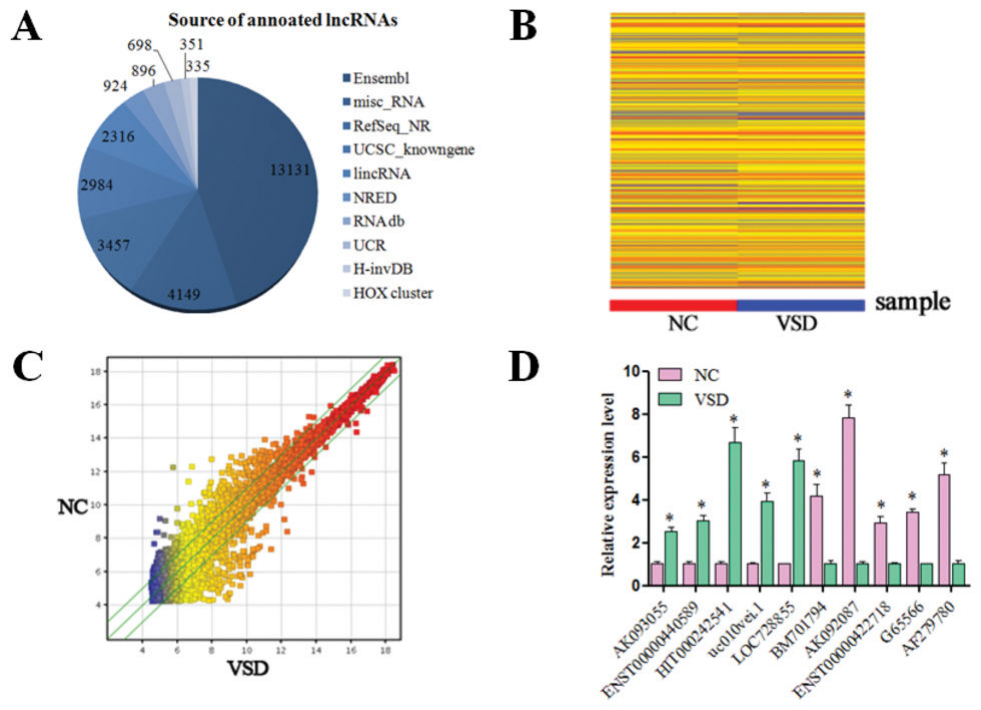
Profile of microarray. (**A**) Microarray v2.0 recovered the vast majority of expressed RefSeq transcripts; 29,241 lncRNAs and 30,215 coding transcripts can be detected using this this microarray. The lncRNAs are carefully collected from the most authoritative databases such as RefSeq, UCSC Knowngenes, Ensembl and related literature. (**B**) The scatterplot is a visualization of the variation (or reproducibility) between chips. (**C**) Hierarchical Clustering was performed to show the distinguishable lncRNAs and mRNA expression pattern among samples. (**D**) Our qRT-PCR data were confirmed to be consistent with the microarray results.

Arraystar Human LncRNA Microarray v2.0 is designed for the global profiling of human lncRNAs and protein-coding transcripts. In total, 29,241 lncRNAs and 30,215 coding transcripts can be detected by this second-generation LncRNA microarray. The lncRNAs are carefully collected from the most authoritative databases such as RefSeq, UCSC Knowngenes, Ensembl and related literature (Figure 1 A). The scatterplot is a visualization that is useful for assessing the variation (or reproducibility) between chips (Figure 1 B). Hierarchical Clustering was performed to show the distinguishable lncRNAs and mRNAs expression pattern among samples (Figure 1 C). We set a threshold as fold-change >2.0 and found that there were 880 upregulated and 628 downregulated lncRNAs in the VSD hearts compared with the NC hearts, thus indicating a difference in the lncRNA expression profiles between the two groups (supplement xls.1 and xls.2). Our microarray analysis data were validated by real-time PCR expression analysis in expanded heart samples of 10 randomly selected differentially expressed lncRNAs with fold-changes >3 (Figure 1 D). Thus, our results indicate that a series of lncRNAs are frequently aberrantly expressed in VSD-affected hearts and that they may be related to the development of this malformation.

### General characteristics of dysregulated lncRNAs in VSD

**Figure 2:**
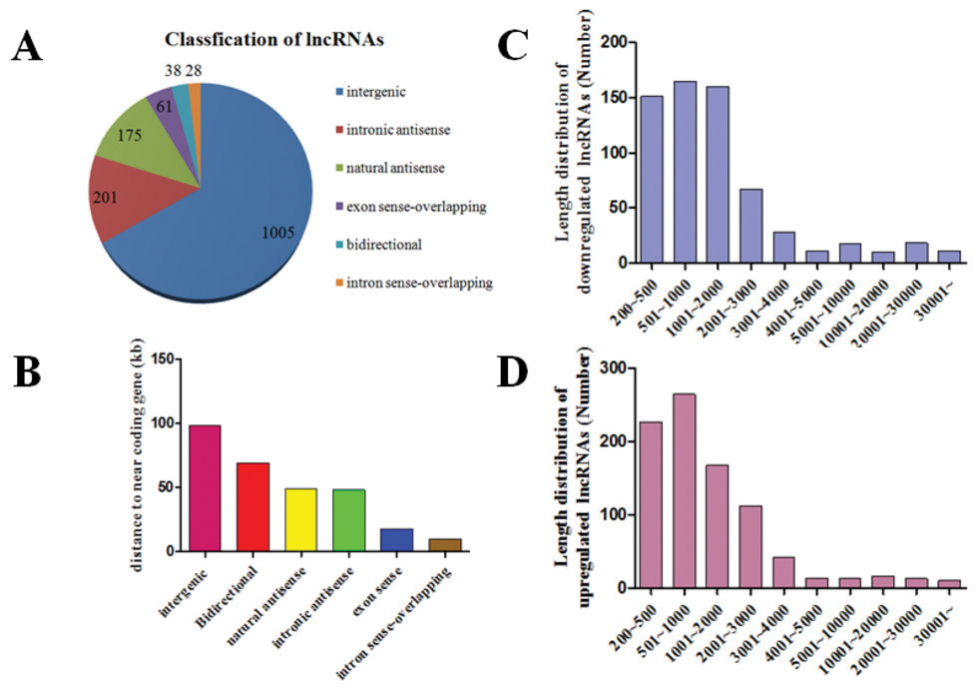
General characteristics of dysregulated lncRNAs in VSD-affected cardiac tissue. (**A**) Among the dysregulated lncRNAs, there were 905 intergenic, 201 intronic antisense, 175 natural antisense, 61 bidirectional, 38 exon sense and 28 intron sense-overlapping sequences. (**B** and **C**) The lncRNAs were mainly between 200 bp and 3000 bp in length. (**D**) In accordance with the order from large to small, the distance between lncRNAs and the closest coding gene is intergenic 98 kb, bidirectional 69 kb, natural antisense 49 kb, intronic antisense 48 kb , exon sense18 kb and intron sense-overlapping 10 kb.

We summarized some general characteristics of dysregulated lncRNAs such as classification, length distribution and distance to neighboring coding genes. Among the dysregulated lncRNAs, there were 905 intergenic, 201 intronic antisense, 175 natural antisense, 61 bidirectional, 38 exon sense and 28 intron sense-overlapping (Figure 2 A). The lncRNAs are mainly between 200 bp and 3000 bp in length (Figure 2 B and C). In accordance with the order from large to small, the distance between lncRNAs and closest coding gene is intergenic 98 kb, bidirectional 69 kb, natural antisense 49 kb, intronic antisense 48 kb, exon sense18 kb and intron sense-overlapping 10 kb (Figure 1 D).

### Go and pathway analysis

**Figure 3:**
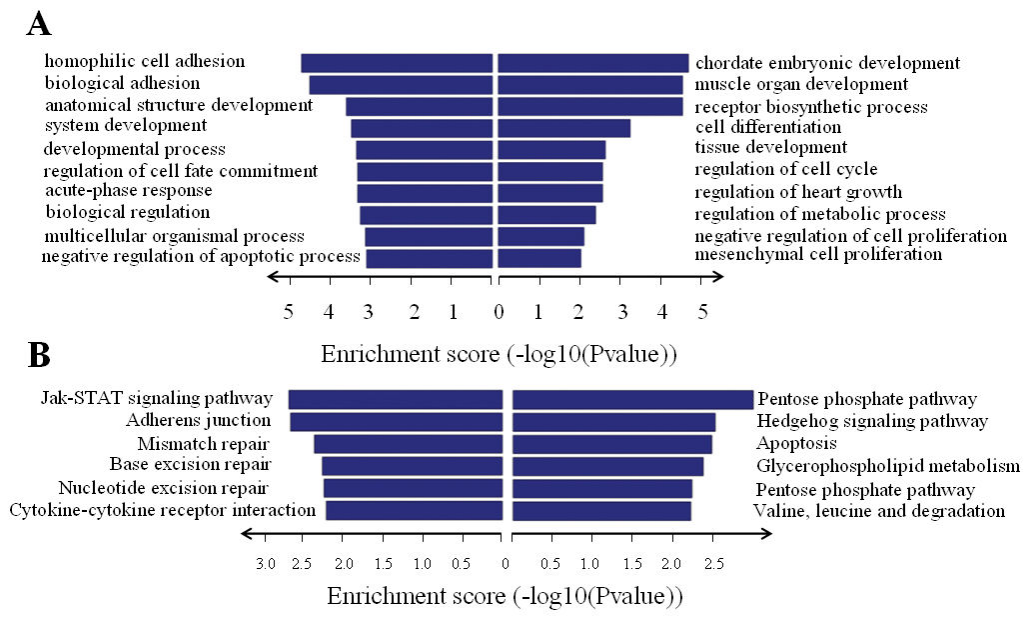
GO and pathway analysis. The first 10 Go ID (**A**) and first six pathways (**B**) that exhibited significant differences between VSD and NC groups are listed (left and Right panel show the associated coding genes of downregulated and upregulated lncRNAs, respectively).

The GO project (*http://www.geneontology.org*) is a collaborative effort to construct and use ontologies to facilitate the biologically meaningful annotation of genes and their products in a wide variety of organisms and is the key functional classification system of the NCBI. In our survey of existing data, the neighbor coding gene function of upregulated lncRNAs mainly involved: (1) GO:0043009: chordate embryonic development; (2) GO:0007517 muscle organ development; (3) GO:0010869 receptor biosynthetic process; (4) GO:0030154 cell differentiation; (5) GO:0060420 regulation of heart growth; (6) GO:0009892 regulation of metabolic process. Meanwhile, the neighbor coding gene function of downregulated lncRNAs mainly involved: (1) GO: 0007156: homophilic cell adhesion; (2) GO:0022610 biological adhesion; (3) GO:0048856: anatomical structure development; (4) GO:0048731: system development; (5) GO:0032502 developmental process; (6) GO:0010453 regulation of cell fate commitments (Figure 3 A).

Ingenuity Pathway Analysis (IPA) was used to identify pathways and gene networks represented among the sets of protein-coding mRNA s identified in the VSD gene expression signature. In our survey of existing data, the neighbor gene function of upregulated lncRNAs mainly involved the following pathways: (1) Pentose phosphate pathway; (2) Hedgehog signaling pathway; (3) Apoptosis; (4) Glycerophospholipid metabolism; (5) Pentose phosphate pathway; (6) Valine, leucine and isoleucine degradation. The neighbor gene function of downregulated lncRNAs mainly involved the following pathways: (1) Jak-STAT signaling pathway; (2) Mismatch repair; (3) Adherens junction; (4) Base excision repair; (5) Nucleotide excision repair; (6) Cytokine-cytokine receptor interaction (Figure 3 B).

### Validation of lncRNAs candidates in VSD

**Figure 4:**
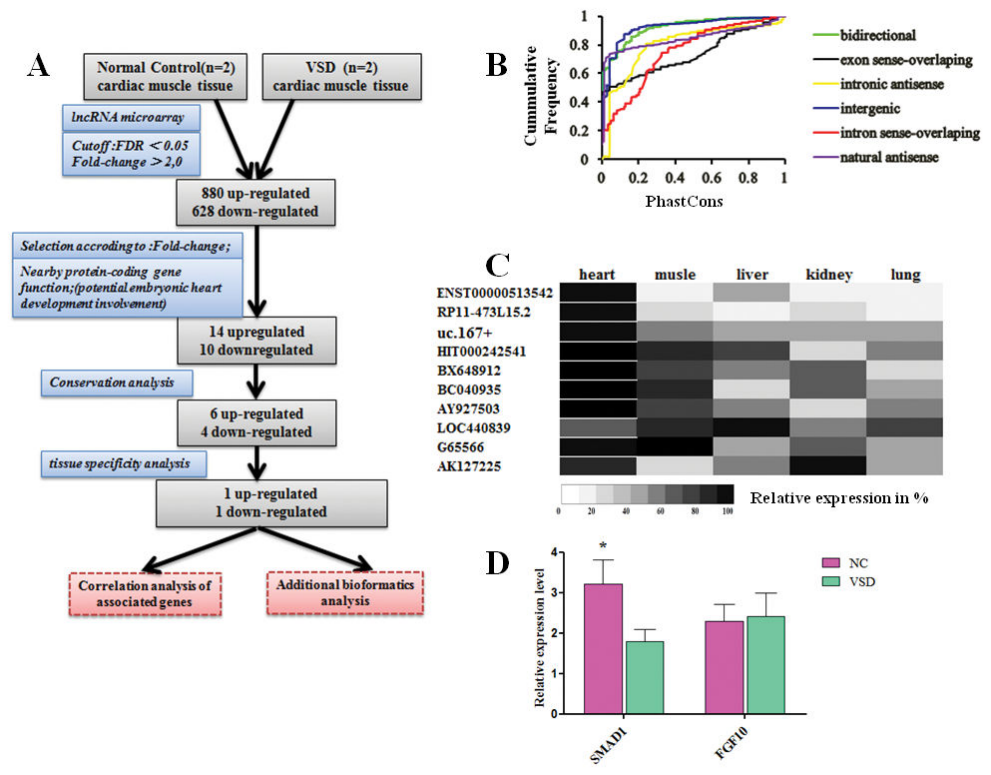
Validation of lncRNA candidates in VSD-affected cardiac tissue. (**A**) The overview of the pipeline for validation of lncRNA candidates in VSD. (**B**) Analysis of the conservation of all classes of dysregulated lncRNAs compared to conservative coding genes revealed an absence of high levels of conservation across species among the majority of lncRNAs. (**C**) The expression of the 10 selected heart expressed lncRNAs transcripts was validated by quantitative PCR in five human fetal tissues. The majority of the tested lncRNAs transcripts are predominately expressed in heart (percentage range; white, 0% to black, 100%). (**D**) To estimate the correlation of these two lncRNAs and the associated genes, we detected SMAD 1 and FGF10 expression in fetal heart tissues. We found that the expression of SMAD1 is significantly lower in VSD heart tissues than NC heart. No significant difference in FGF10 expression was observed between the two groups.

The overview of the pipeline for validation of lncRNA candidates in VSD is shown in (Figure 4 A). For practical purposes, to reduce the lncRNAs for further investigation and to enrich those potentially involved in VSD, we first selected candidate transcripts with an expression fold-change >2 that were associated with an annotated protein-coding gene where a biological function in myocardial cells has been proposed according to the scientific literature and through GO term enrichment of the following processes: “regulation of heart growth ” (*P* = 0.008), “regulation of cell cycle” (*P* = 0.007), “cell differentiation” (*P* = 0.004), “regulation of cell fate commitment” (*P* < 0.001), “negative regulation of apoptotic process” (*P* < 0.001) and “developmental process ” (*P* < 0.001). The selected lncRNAs are listed in Table S2.

Next, we analyzed the conservation of all classes of dysregulated lncRNAs, compared to conservative coding genes; the majority of lncRNAs did not have high conservation across species (Figure 4 B). Moreover we calculated the base conservation in each lncRNA using the criteria that the phastCons score of base is not less than 0.5 indicates high conservation. By applying these two steps, we selected six upregulated and four downregulated lncRNAs which are not only potentially involved in heart development but are also conserved across species.

Finally, we validated the expression of the 10 selected cardiac lncRNAs transcripts by quantitative PCR in five human fetal tissues. These tissues were chosen because they show different degrees of cell type complexity and biological functionality. We found that the large majority of the tested lncRNA transcripts are predominately expressed in heart tissue (Figure 4 C). According to relative expression, we selected ENST00000513542 and RP11-473L15.2 for further analysis. Their associated genes are SMAD1 and FGF10, respectively. We then detected their expression in fetal heart tissue to estimate the correlation of these two lncRNAs and their associated genes. It was found that the expression of SMAD1 was significantly lower in VSD-affected heart tissues than in NC heart. However, there was no significant difference in FGF10 expression between two groups (Figure 4 D).

### Bioinformatics analysis of lncRNA ENST00000513542 and RP11-473L15.2

**Figure 5:**
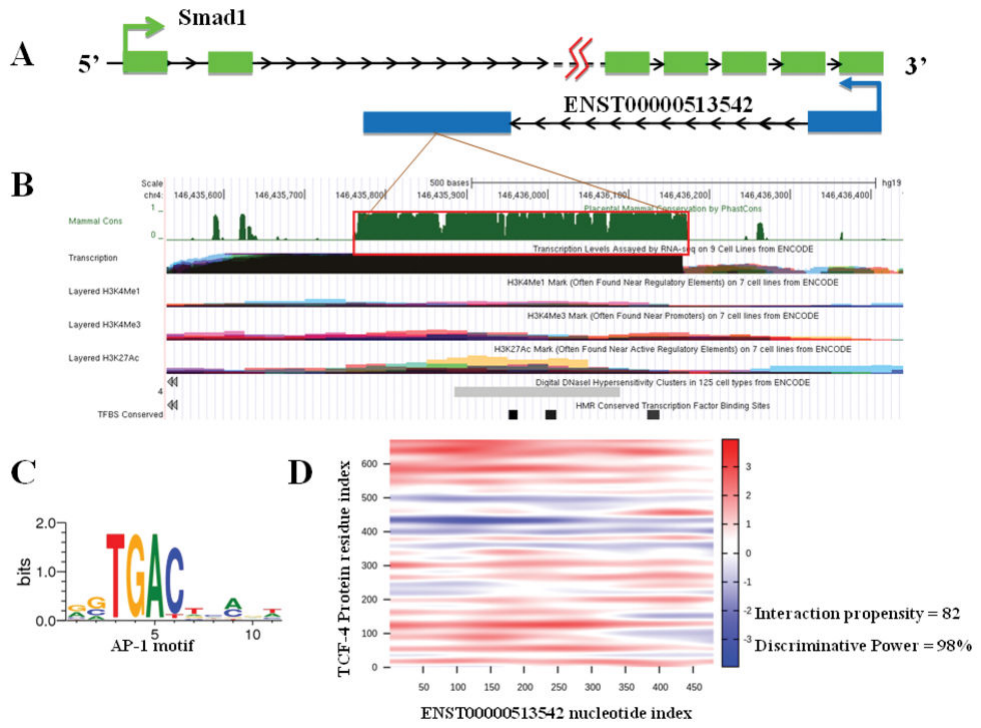
Bioinformatics analysis of ENST00000513542. (**A**) ENST00000513542 is a natural antisense lncRNA, transcribed from 2,105 bp downstream of the second exon of the *Smad1* gene. (**B**) Several tracks of interest, including conservation, histone markings, DNase hypersensitivity, and TFBS are displayed. Integrated Regulation track data for a region spanning the ENST0000051354 are shown. The red box shows a region of overlap between the various tracks. (**C**) Transcription factor binding sites (TFBS) prediction indicated that ENST0000051354 loci combine with AP-1 as a cis-acting element. (**D**) Further catRAPID analysis indicated a strong RNA-protein interaction between ENST0000051354 and TCF-4, which is the TF of SMAD1.

ENST00000513542 is a natural antisense lncRNA transcribed from 2,105 bp downstream of the second exon of the *Smad1* gene (Figure 5 A). The Integrated Regulation track is actually six separate tracks, collectively referred to as a “super-track.” Thus, the “DNase Clusters” track summarizes DNase hypersensitivity mapping, the “Txn Factor ChIP” track summarizes transcription factor mapping, and the “Layered H3K4me1, H3K4me3, H3K27ac” tracks summarize covalent histone modification mapping. The red box shows Integrated Regulation track data for a region spanning the human lncRNA (Figure 5 B). Prediction of TFBS (transcription factor binding sites) indicated that ENST0000051354 loci combine with an AP-1 motif as a cis-acting element (Figure 5 C). Further catRAPID analysis indicated a strong RNA-protein interaction between ENST0000051354 and TCF-4, a TF of SMAD1 (Figure 5 D).

RP11-473L15.2 is a bidirectional lncRNA, 49 bp from the transcription start site of FGF10 (Figure S1 A). As for Figure 5, we identified a region of overlap between the various tracks in the RP11-473L15.2 loci (Figure S1 B). Prediction of TFBS indicated that RP11-473L15.2 combines with the SRF (serum response factor) motif (Figure S1 C). However, no intense RNA-protein interactions were identified between RP11-473L15.2 and TFs of FGF10 (Figure S1 D).

## Discussion

In the present study, by comparing the transcriptome profiles of VSD-affected cardiac and normal tissues using the microarray technique, we have generated an integrated analysis of dysregulated lncRNAs, focusing specifically on the identification and characterization of potential lncRNAs involved in the initiation of VSD. We recovered the vast majority of expressed RefSeq transcripts; 3,045 lncRNAs and 30,215 coding transcripts were detectable using this second-generation LncRNA microarray. We identified 880 upregulated and 628 downregulated lncRNAs, and summarized their general characteristics. Subsequently, through our established filtering pipeline, we selected two lncRNAs, ENST00000513542 and RP11-473L15.2, which may be closely related to VSD. Thus, our data set provides a comprehensive profile and analysis of lncRNAs transcripts in human fetal heart with VSD.

The Gene Ontology project provides a controlled vocabulary to describe gene and gene product attributes in any organism [14]. In our survey of existing data, the main biological processes involving dysregulated lncRNAs included many closely connected to heart development, such as “regulation of cell cycle” “cell differentiation” “regulation of cell fate commitment” “developmental process” and “regulation of cell fate commitment”. However, perhaps the most important current challenge is that the knowledge embedded in pathways regarding how various genes interact with each other is not currently exploited. Microarray technology makes it possible to measure the expression levels of almost all the human genes and therefore facilitates the identification of genes and pathways that are related to disease initiation and development. Based on our data, the neighbor coding gene of dysregulated lncRNAs in VSD mainly involved pathways involved in DNA damage-repair, energy metabolism and apoptosis. For instance, the JAK (Janus kinase)-STAT (Signal Transducer and Activator of Transcription) signaling pathway plays an important role in transmitting information from extracellular polypeptide signals to target gene promoters in the nucleus. JAK-STAT signaling regulates many cellular processes including development, cell proliferation, differentiation, and apoptosis[19].

 Due to the limited information available about the function of each lncRNA, we applied several additional filtering steps to isolate the most robust transcripts. First, we selected only those whose higher fold expression change overlapped with protein-coding genes with functions related to heart development or cell proliferation, apoptosis and differentiation. Second, we selected only the conservative lncRNAs, since conservation is thought to indicate sequences that are functionally important. Although the entire transcripts of most long ncRNAs lack strong conservation, highly conserved elements are preserved in these sequences. On the other hand, most protein-coding genes are expressed in multiple tissues [20]. In contrast, lncRNA expression tends to be spatially and temporally restricted[21,22]. Tissue-specificity of lncRNAs was previously based on differential expression patterns in specific biological systems. In this study, the majority of lncRNAs we identified were also remarkably tissue-specific compared with protein-coding genes. It is consistent with the hypothesis that some lncRNAs interact with chromatin modulators and provide their target specificity, as well as it may indicate that lncRNAs could serve as specific fine-tuners[23].

One of the main challenges for lncRNA research is the identification of an association with a particular molecular or cellular function. One possibility is that lncRNAs act locally, regulating the expression levels of neighboring RNA transcripts[24]. Herein, we have studied the possible effect of ENST00000513542 and RP11-473L15.2 on the activity of their associated protein-coding genes (*Smad1* and FGF10, respectively). We found that expression of Smad1 significantly decreased in VSD-affected heart compared to NC, which indicates that ENST00000513542 is involved in cis-regulation of the *Smad1* target gene. The SMAD1 protein is a signal transducer and transcriptional modulator that mediates multiple signaling pathways. This protein mediates the signals of the bone morphogenetic proteins (BMPs), which are involved in a range of biological activities including cell growth, apoptosis, morphogenesis, heart development and immune responses. In contrast, there was no difference in FGF10 expression between the two groups, which may indicate that RP11-473L15.2 does not affect FGF10 expression at transcriptional level.

The language used by lncRNAs to interact with network components is still largely elusive. Unlike the well-studied miRNAs, lncRNAs do not seem to function via a common pathway; therefore, no predictions can be made about their function based on their primary sequence or secondary structure. A major challenge lies in decoding the functional elements and modules in the primary sequence of non-coding genes, including the structural motifs and regulatory elements that define their roles[25]. Currently, there are no features of either the genome or epigenome that can be used to unequivocally identify regulatory elements. Nevertheless, some features, such as DNase hypersensitivity, transcription factor occupancy, and histone modifications, seem to be more reliable indicators of regulatory function than others[26]. Based on our analysis, for ENST0000051354 or RP11-473L15.2, we identified a region of overlap between the various tracks, which indicated that these lncRNAs function as regulatory elements. Chromatin immunoprecipitation experiments followed by deep-sequencing have revealed that many lncRNAs show specific binding at their promoters by key developmental transcription factors[27]. In this study, TFBS prediction indicated that ENST0000051354 combines with activator protein 1 (AP-1), while RP11-473L15.2 may combine with serum response factor (SRF). However, critical roles for AP-1 or SRF as TFs in the regulation of cell proliferation and differentiation remain to be confirmed. The genomic targets of AP-1 and SRF in cardiomyocytes show the importance of combinatorial transcription factor regulation and that ncRNAs form an important component of the cardiac gene regulatory network. Further catRAPID analysis indicated that there is a strong RNA-protein interaction between ENST0000051354 and TCF-4, a TF of SMAD1, which further indicated its functional importance. Nevertheless, identification of their function requires future loss-of-function or gain-of-function analysis.

Finally, it should be mentioned that the remaining lncRNAs we did not select for further analysis should not be taken as evidence of no biological relevance. Although extreme evolutionary sequence conservation has been used to identify functional elements, many lncRNAs, including those functional in heart, are in fact poorly conserved [28]. The mere physical proximity of lncRNAs and genes with developmental functions does not necessarily imply a functional link between the protein-coding gene and the lncRNA [29]. For example, recent studies in the mouse did not detect a strong correlation between the expression levels of most lncRNAs and their neighbors [30]. One important reason that may account for this is that lncRNAs may employ varied mechanisms of action; they do not always function in cis- or in trans.

In summary, for the first time our study provides a profile of dysregulated lncRNAs in human fetal heart tissues with VSD. It suggests numerous lncRNAs are involved in heart development and provides a background/reference resource for future functional investigations of lncRNAs related to VSD. Further studies are necessary to reveal possible biological functions and molecular mechanisms exerted by these lncRNAs in the process of VSD formation.

## Supporting Information

Table S1 **Primers used in this study.**
(DOC)

Table S2 **LncRNAs implicated in heart development.**
(DOC)

Figure S1 **Bioinformatics analysis of RP11-473L15.2.** (**A**) RP11-473L15.2 is a bidirectional lncRNA, located 49 bp from the transcription start site of FGF10. (**B**) As for Figure 5, a region of overlap between the various tracks in RP11-473L15.2 loci was also identified. (**C**) Prediction of TFBS indicated that RP11-473L15.2 combines with SRF. (**D**) However, no intense RNA-protein interaction between RP11-473L15.2 and TFs of FGF10 was identified.
(TIF)
