# Supplementary material for: Integrated Analysis of Dysregulated lncRNA Expression in Fetal Cardiac Tissues with Ventricular Septal Defect
Source: PLoS One. 2013 Oct 16;8(10):e77492. doi: 10.1371/journal.pone.0077492 (PMC3797806; doi:10.1371/journal.pone.0077492)
Supplement: Table S1 — Primers used in this study. (DOC) [file pone.0077492.s001.doc]

**Table S1.** **Primers used in this study.**

| **Gene name** | **Forward (5’ – 3’)** | **Reverse (5’ – 3’)** |
| --- | --- | --- |
| AK093055 | AAATAGTTGAAAGCGGTGTA | CCTGTCTGGGCTGTAGTG |
| GAPDH | GACCTGACCTGCCGTCTA | AGGAGTGGGTGTCGCTGT |
| ENST00000440589 | ACTTGGAACTATGGGAGG | GGGAGAATTAAAGGCTAAA |
| HIT000242541 | GCCGCTGGCTCTTAGTGT | CCTTGGAGGGAGGGATTAG |
| uc010vei.1 | GGGTTTGAATGAGATGAGGG | AGTTCTGGAATGGAAGGGTT |
| LOC728855 | GCGGATGACAGACACGGAGAT | GGGCAAAGGAGGGCAAGT |
| BM701794 | CGCCTGTAATCCCAGCAC | GGGTTCACGCCATTCTCC |
| AK092087 | ACTTCCTGCCCTGGTTCA | ATGCCTTCTCCTGGTGCT |
| ENST00000422718 | ATAATCAACTTCCTAACCGTAC | CACCTTCACAGGCTTTCT |
| G65566 | ACGACCAGCGGCTTAGAG | TTTCCTGTTGGGCTCACG |
| AF279780 | ACTGCCTACTTGTATGTCA | TCTATTTGCTGGTCGTGT |
| RP11-473L15.2 | TCGTCGTCATTAAGCATT | GAAGATCACAGAGGAGCA |
| ENST00000513542 | AGATGAAATGCTGGAAGTG | CAGAGCCACCATGAACTAA |
| uc.167+ | ACCCCTCCAGTCTCAACT | CCCACCGTAAAACTAAAA |
| HIT000242541 | GCCGCTGGCTCTTAGTGT | CCTTGGAGGGAGGGATTAG |
| BX648912 | CACGAGGCAGTAGAAACC | CAGGGATGGATAAGACAGAT |
| BC040935 | CAGTCCAGGTCAAGTTCC | GCTTACAGTTTGTGGGAGA |
| AY927503 | TGCCATCAACAGAACATCA | CAGGAGGAGCAGCCATAC |
| LOC440839 | GAAAGAAGTCATACTGTGGGTA | CAATGTTGATGGGTGCTG |
| AK127225 | GAGTCCAAAGGCCGAAGA | CACAAAGGGAGAAGACAAGC |
| FGF10 | CCACCAACTCTTCTTCCT | TCCCGTTCTTCTCAATCT |
| Smad1 | AGTTCTTACTCAAATGGGTTCA | AGGCTCCTTTGTCAGTTCTC |
