# Supplementary material for: Integrated Analysis of Dysregulated lncRNA Expression in Fetal Cardiac Tissues with Ventricular Septal Defect
Source: PLoS One. 2013 Oct 16;8(10):e77492. doi: 10.1371/journal.pone.0077492 (PMC3797806; doi:10.1371/journal.pone.0077492)
Supplement: Table S2 — LncRNAs implicated in heart development. (DOC) [file pone.0077492.s002.doc]

**Table S2. LncRNAs implicated in heart development**

|  | **LncRNA** | **Length** | **Source** | **Associated Gene** | **Relationship** |
| --- | --- | --- | --- | --- | --- |
| **downregulated** | uc.299- | 210 | UCR | PAX2 | natural antisense |
|  | NR_024156 | 4929 | RefSeq_NR | SYK | exon ense-overlapping |
|  | AK092789 | 2015 | RNAdb | H2AFY | bidirectional |
|  | AK127225 | 4949 | misc_RNA | ZNF7 | bidirectional |
|  | uc.329- | 307 | UCR | WT1 | intergenic |
|  | nc-HOXA13-96 | 318 | HOX cluster | HOXA13 | bidirectional |
|  | NR_026593 | 1745 | RefSeq_NR | MED19 | bidirectional |
|  | NR_029399 | 1188 | RefSeq_NR | PAX8 | Intron sense-overlapping |
|  | RP11-473L15.2 | 1894 | Ensembl | FGF10 | bidirectional |
|  | AK309872 | 1463 | NRED | ATP1A1 | natural antisense |
| **upregulated** | BC040935 | 833 | UCSC_knowngene | MCF2L2 | intronic antisense |
|  | ENST00000500011 | 2272 | Ensembl | KIAA0913 | natural antisense |
|  | ENST00000451530 | 781 | Ensembl | NRP1 | natural antisense |
|  | HIT000092072 | 1145 | H-invDB | GPR150 | natural antisense |
|  | nc-HOXA5-67 | 226 | HOX cluster | HOXA5 | bidirectional |
|  | AY927503 | 906 | RNAdb | ZNF282 | natural antisense |
|  | uc010vei.1 | 394 | UCSC_knowngene | MAPK3 | natural antisense |
|  | BC085014 | 819 | UCSC_knowngene | RASA4 | natural antisense |
|  | HIT000054536 | 4569 | H-invDB | ABLIM3 | natural antisense |
|  | nc-HOXC6-244 | 1488 | HOX cluster | HOXC4 | intron sense-overlapping |
|  | BX648912 | 2619 | lincRNA | GJB5 | exon sense-overlapping |
|  | nc-HOXC11-116 | 228 | HOX cluster | HOXC11 | bidirectional |
|  | ENST00000513542 | 481 | Ensembl | SMAD1 | natural antisense |
|  | HIT000242541 | 862 | H-invDB | DUSP6 | natural antisense |
