# Supplementary figures and images for: Integrated Analysis of Dysregulated lncRNA Expression in Fetal Cardiac Tissues with Ventricular Septal Defect
Source: PLoS One. 2013 Oct 16;8(10):e77492. doi: 10.1371/journal.pone.0077492 (PMC3797806; doi:10.1371/journal.pone.0077492)

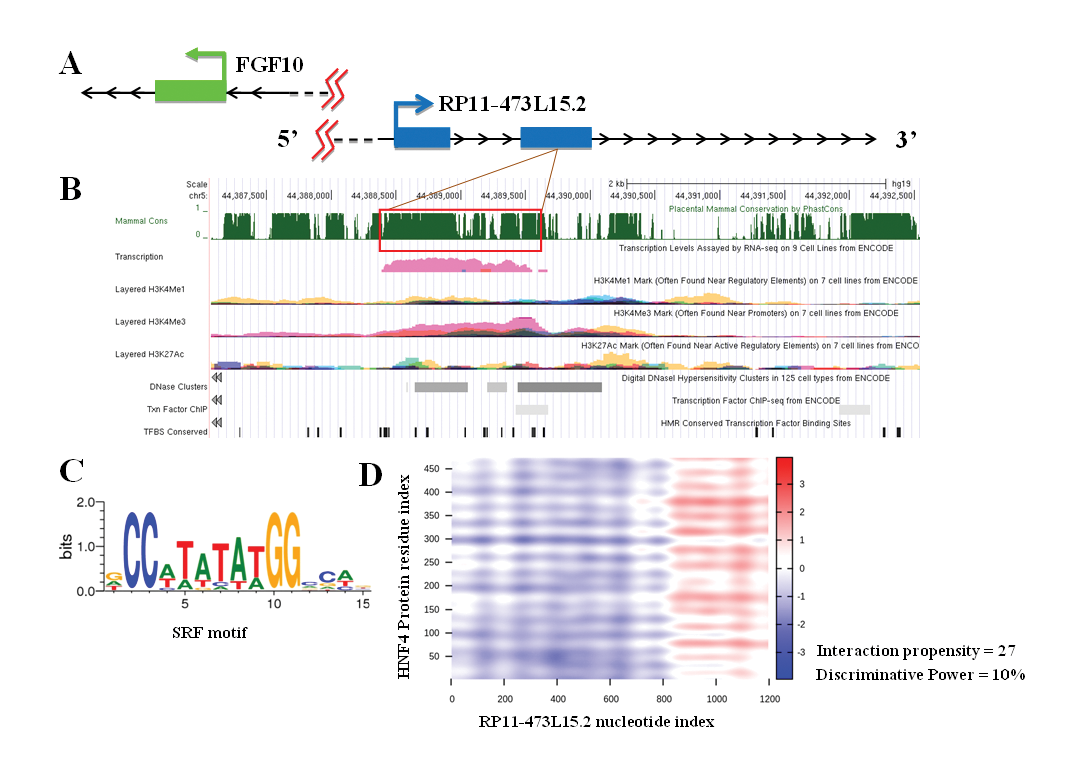

Supplement: Figure S1 — Bioinformatics analysis of RP11-473L15.2. (A) RP11-473L15.2 is a bidirectional lncRNA, located 49 bp from the transcription start site of FGF10. (B) As for Figure 5, a region of overlap between the various tracks in RP11-473L15.2 loci was also identified. (C) Prediction of TFBS indicated that RP11-473L15.2 combines with SRF. (D) However, no intense RNA-protein interaction between RP11-473L15.2 and TFs of FGF10 was identified. (TIF) [file pone.0077492.s003.tif]
